# Vascular complications and outcomes following transcatheter aortic valve replacement in patients on chronic steroid therapy: a meta-analysis

**DOI:** 10.1097/JS9.0000000000001132

**Published:** 2024-02-05

**Authors:** Song Peng Ang, Jia Ee Chia, Vikash Jaiswal, Muhammed Hanif, Ananya Vadhera, Sudarshan Gautam, Anuradha Raut, Saira Rafaqat, Vamsikalyan Reddy Borra, Harshwardhan Khandait, Abhigan Babu Shrestha, Jose Iglesias

**Affiliations:** a Department of Internal Medicine, Rutgers Health/Community Medical Center, Toms River, NJ, USA; b Department of Critical Care, Rutgers Health/Community Medical Center, Toms River, NJ, USA; c Department of Internal Medicine, Trinitas Regional Medical Center/RWJ Barnabas Health, Elizabeth, NJ, USA; d Department of Internal Medicine, SUNY Upstate Medical University, Syracuse, NY, USA; e Department of Internal Medicine, University of Texas Rio Grande Valley, Edinburg, TX, USA; f Department of Internal Medicine, Maimonides Medical Center, Brooklyn NY, USA; g Department of Internal Medicine, Nepal Medical College, Nepal; h Department of Zoology (Molecular Physiology), Lahore College for Women University, Lahore, Pakistan; i JCCR Cardiology Research, Varanasi, India; j Department of Medicine, Maulana Azad Medical College, New Delhi, India; k Department of Internal Medicine, M Abdur Rahim Medical College, Dinajpur, Bangladesh; l Department of Medicine, International Medical University, Malaysia

**Keywords:** aortic stenosis, outcomes, steroids, TAVR

## Abstract

**Background:**

Chronic steroid (CS) therapy was reportedly linked to increased vascular complications following percutaneous coronary intervention. However, its association with vascular complications after transcatheter aortic valve replacement (TAVR) remained uncertain, with conflicting results being reported.

**Objective:**

The authors aimed to compare the rate of vascular complications and outcomes between patients with and without CS use after TAVR.

**Methods:**

The authors conducted a comprehensive literature search in PubMed, Embase, and Cochrane databases from their inception until 18th April 2022 for relevant studies. Endpoints were described according to Valve Academic Research Consortium-2 definitions. Effect sizes were pooled using DerSimonian and Laird random-effects model as risk ratio (RR) with 95% CI.

**Results:**

Five studies with 6136 patients undergoing TAVR were included in the analysis. The included studies were published between 2015 and 2022. The mean ages of patients in both study groups were similar, with the CS group averaging 80 years and the nonsteroid group averaging 82 years. Notably, a higher proportion of patients in the CS group were female (56%) compared to the nonsteroid group (54%). CS use was associated with a significantly higher risk of major vascular complications (12.5 vs. 6.7%, RR 2.32, 95% CI: 1.73–3.11, *P*<0.001), major bleeding (16.8 vs. 13.1%, RR 1.61, 95% CI: 1.27–2.05, *P*<0.001), and aortic annulus rupture (2.3 vs. 0.6%, RR 4.66, 95% CI: 1.67–13.01, *P*<0.001). There was no significant difference in terms of minor vascular complications (RR 1.43, 95% CI: 1.00–2.04, *P*=0.05), in-hospital mortality (2.3 vs. 1.4%, RR 1.86, 95% CI: 0.74–4.70, *P*=0.19), and 30-day mortality (2.9 vs. 3.1%, RR 1.14, 95% CI: 0.53–2.46, *P*=0.74) between both groups.

**Conclusion:**

Our study showed that CS therapy is associated with increased major vascular complications, major bleeding, and annulus rupture following TAVR. Further large multicenter studies or randomized controlled trials are warranted to validate these findings.

## Introduction

Highlights

- The management of patients on chronic steroid therapy requiring transcatheter aortic valve replacement (TAVR) poses a unique clinical challenge.
- The concerns surrounding potential complications arise from the use of immunosuppressive agents.
- Our study showed that chronic steroid therapy is associated with increased major vascular complications, major bleeding, and annulus rupture following TAVR.

Transcatheter aortic valve replacement (TAVR) has revolutionized the treatment of severe aortic stenosis, offering a less invasive alternative to surgical aortic valve replacement, particularly in patients considered high-risk for conventional surgery^1^. Over the recent years, TAVR has become the standard of care for these patients, with substantial evidence supporting its efficacy and safety^2,3^. However, the management of patients on chronic steroid (CS) therapy requiring TAVR poses a unique clinical challenge due to concerns surrounding potential complications arising from the use of immunosuppressive agents^4,5^. Findings from percutaneous coronary interventions have revealed a notable rise in the incidence of complications among these individuals^6^. CS use is prevalent in various inflammatory, autoimmune, and allergic conditions, and its impact on TAVR outcomes remains a subject of active investigation. Understanding the potential risks and benefits of TAVR in patients on CS therapy compared to those without is vital to inform clinical decision-making and optimize patient care. In this study, we aim to present a comprehensive comparative analysis of outcomes following TAVR in these distinct patient populations, shedding light on the implications of CS therapy on procedural success, postoperative complications, and survival. Our findings have the potential to improve the current understanding and enhance the overall management of patients with severe aortic stenosis requiring TAVR in the context of CS therapy.

## Methods

This systematic review was conducted and reported following the Cochrane and PRISMA (Preferred Reporting Items for Systematic Review and Meta-analysis) (Supplemental Digital Content 1, http://links.lww.com/JS9/B798, Supplemental Digital Content 2, http://links.lww.com/JS9/B799) 2020 guidelines as described previously^7–9^. A prespecified study protocol has been registered in the PROSPERO.

### Search strategy

We conducted a systematic search in PubMed, Embase, Scopus, and Cochrane Central for articles from their inception until 15th September 2023, using the following keywords and MeSH Terms: (‘Aortic Stenosis’[Mesh] OR ‘Heart Valve Diseases’[Mesh] OR ‘Aortic Valve’[Mesh]) AND (‘Transcatheter Aortic Valve Replacement’[Mesh] OR ‘Transcatheter Aortic Valve Implantation’[Mesh] OR ‘TAVR’[All Fields] OR ‘TAVI’[All Fields]) AND (‘Steroids’[Mesh] OR ‘Corticosteroids’[Mesh]).

### Study selection

Studies that were included had all the following parameters: i) patients who underwent TAVR, ii) studies with patients >18 years, iii) two-arm studies in which the intervention group is all patients who received corticosteroids and another arm being patients who did not receive corticosteroids, iv) studies reporting at least one of the desired outcomes and v) studies such as prospective, and retrospective were sought to be eligible. We excluded literature or systematic reviews, letters, studies with single-arm, animal studies, and studies with patients <18 years of age. Two authors (S.P.A. and J.E.C.) independently examined each potential paper identified through comprehensive electronic database searches and manual screening of relevant articles. Any discrepancies between the two authors regarding the inclusion of a study were resolved by a third author (V.J.) through consensus. Studies were included if they met the predefined eligibility criteria, which encompassed TAVR outcomes in patients on CS therapy compared to those without.

### Study outcomes

The primary outcomes of this meta-analysis were major vascular complications and major bleeding. The secondary outcomes were aortic annulus rupture, cardiac tamponade, minor vascular complications, stroke, in-hospital mortality, 30 days mortality, and pacemaker implantation. Major and minor vascular complications were defined according to the Valve Academic Research Consortium-2 consensus criteria (VARC-2)^10^. Major vascular complications, as defined by the VARC-2 criteria, include access-related nerve or vascular injuries leading to outcomes such as death, major bleeding, or visceral ischemia, as well as significant aortic injuries encompassing aortic dissection, rupture, and annulus rupture. This category also covers noncerebral distal embolization and scenarios necessitating unplanned surgical or endovascular interventions that are associated with major bleeding, ischemia, or death. In contrast, minor vascular complications are characterized by access-site vascular injuries, noncerebral distal embolization, or unplanned interventions, provided these do not result in death, major bleeding, ischemia, or neurological damage^10^.

### Data extraction and statistical analysis

Data from the eligible studies, such as demographic, study design, comorbidity, follow-up, and clinical outcomes post-TAVR among both groups of patients were extracted. To ensure the integrity and validity of our analysis, we took stringent measures to identify and exclude any duplicated patient populations or overlapping data within the selected studies. Two authors (S.P.A. and J.E.C.) independently reviewed the patient characteristics and clinical data reported in each included study. Any instances of overlapping data or identical patient cohorts across multiple studies were meticulously identified. Baseline continuous variables were summarized in mean (SD), whereas dichotomous variables were described in frequency or percentage. We performed a conventional meta-analysis for primary and secondary outcomes and adopted the DerSimonian and Laird random-effect model for the study variations^11^. Outcomes were reported as pooled risk ratio (RR), standard mean difference (SMD), and their corresponding 95% CI. Statistical significance was met if the 95% CI did not cross the numeric ‘1’ and the two-tailed *P*-value was less than 0.05. We considered a two-tailed *P*-value of less than 0.05 to be statistically significant. In addition, we assessed the between-study heterogeneity using the Higgins I-square (*I* ^2^) test, with *I* ^2^ values <75% considered mild-moderate and >75% considered high^12^. Sensitivity analysis was performed using a leave-one-out method for outcomes with at least five studies to test the robustness of the primary analysis. Assessment of publication bias was performed for outcomes with at least five studies using funnel plots as well as Egger’s regression test^13^. All statistical work, inclusive analysis, and graphical illustrations were conducted using STATA (version 17.0, StataCorp)^14^.

### Quality assessment

VJ independently assessed the quality of the included studies using the Newcastle–Ottawa Scale for cohort studies^15^.

## Results

The preliminary database search using the prespecified keywords yielded 217 articles. Of these, 142 duplicate studies were excluded, and 46 studies were further excluded from the initial post-title and abstract screening based on the inclusion and exclusion criteria and comparison arm. The full-text review was conducted for the remaining 29 studies. Of these, 24 studies were excluded as they either had the wrong or overlapped populations, lack of outcomes, or were revied articles or case reports. Hence, five studies that met the eligibility criteria were included in our study^4,16–19^. The Preferred Reporting Items for Systematic Reviews and Meta-Analyses (PRISMA) flow diagram is depicted in Supplementary Figure 1 (Supplemental Digital Content 3, http://links.lww.com/JS9/B800). The quality assessment of the included studies was a low to moderate risk on NOS for all studies on bias assessment (Supplementary Table 1, Supplemental Digital Content 4, http://links.lww.com/JS9/B801).

### Baseline patient demographics

A total of 6136 patients were included in our analysis. The ages of patients in both groups were comparable: the mean age of patients in the corticosteroid (CS) group was 80 years, whereas the mean age in the nonsteroid group was 82 years. The number of females in the CS group was slightly higher compared with the nonsteroid group (56 vs 54%). Comorbidities including DM (30 vs. 27%) and HTN (84 vs. 85%) were comparable between both groups of patients. Likewise, the use of balloon-expandable valves (62 vs 62%) and self-expanding valves (29.0 vs 31.5%) were similar between both group of patients. Majority of patients underwent TAVR via the transfemoral route. Characteristics of included studies and patients are presented in Table 1.

**Table 1:** Baseline demographics, comorbidities, and study characteristics of studies included in the meta-analysis.

Variables	Gautier *et al*.^17^	Joshi *et al*.^4^	Koyama *et al*.^15^	Fink *et al*.^16^	Bernhard *et al*.^18^
Total sample					
1. Steroid	48	99	67	25	89
2. Nonsteroid	1251	992	1246	195	2124
Study type	Prospective	Retrospective	Prospective	Retrospective	Prospective
Age (Mean)					
1. Steroid	80	80.2	80.9	78	80.4
2. Nonsteroid	81	81.6	84.6	81	82.1
Age (SD)					
1. Steroid	10	7.4	6	10	6.8
2. Nonsteroid	9	8.1	5	8	6
Female					
1. Steroid	29	51	52	13	39
2. Nonsteroid	589	436	895	109	1101
BMI (Mean)			–		–
1. Steroid	–	27.5		27	
2. Nonsteroid		28.6		27	
BMI (SD)	–		–		–
1. Steroid		5.3		4	
2. Nonsteroid		7.7		5	
Smoking	–				–
1. Steroid		5	12	3	
2. Nonsteroid		40	241	26	
CAD		–			
1. Steroid	15		18	10	51
2. Nonsteroid	455		335	88	1312
Hypertension	–				
1. Steroid		89	48	21	77
2. Nonsteroid		896	969	173	1818
Diabetes mellitus					
1. Steroid	10	25	25	10	28
2. Nonsteroid	322	334	317	71	551
COPD		–			
1. Steroid	10		9	13	15
2. Nonsteroid	185		219	36	263
CKD					–
1. Steroid	–	1	36	13	
2. Nonsteroid		26	732	48	
Euroscore					–
1. Steroid	4.6 (3.6–6.9)	–	4.0 (2.6–7.0)	7.38	
2. Nonsteroid	4.7 (2.8–7.6)		3.6 (2.2–5.7)	5.51	
EURO (Mean)		–			–
1. Steroid	5.03		4.53	7.38	
2. Nonsteroid	5.03		3.83	5.51	
STS risk					
1. Steroid	–	10.56 +/- 7.52	7.1 (4.7–11.0)	6.59	6.23 +/- 6.58
2. Nonsteroid		9.86 +/- 7.55	6.5 (4.6–9.2)	5.36	5.41+/- 3.97
STS (Mean)	–				
1. Steroid		10.56	7.6	6.59	6.23
2. Nonsteroid		9.86	6.77	5.36	5.41
Balloon-expandable					
1. Steroid	26	66	63	–	49
2. Nonsteroid	754	749	1117		1026
Self-expanding					
1. Steroid	21	33	4	13	37
2. Nonsteroid	485	239	129	135	976
Transfemoral access					
1. Steroid	41	99	67	25	–
2. Nonsteroid	1018	992	1246	195	

### Primary and secondary outcomes

**Figure 1:**
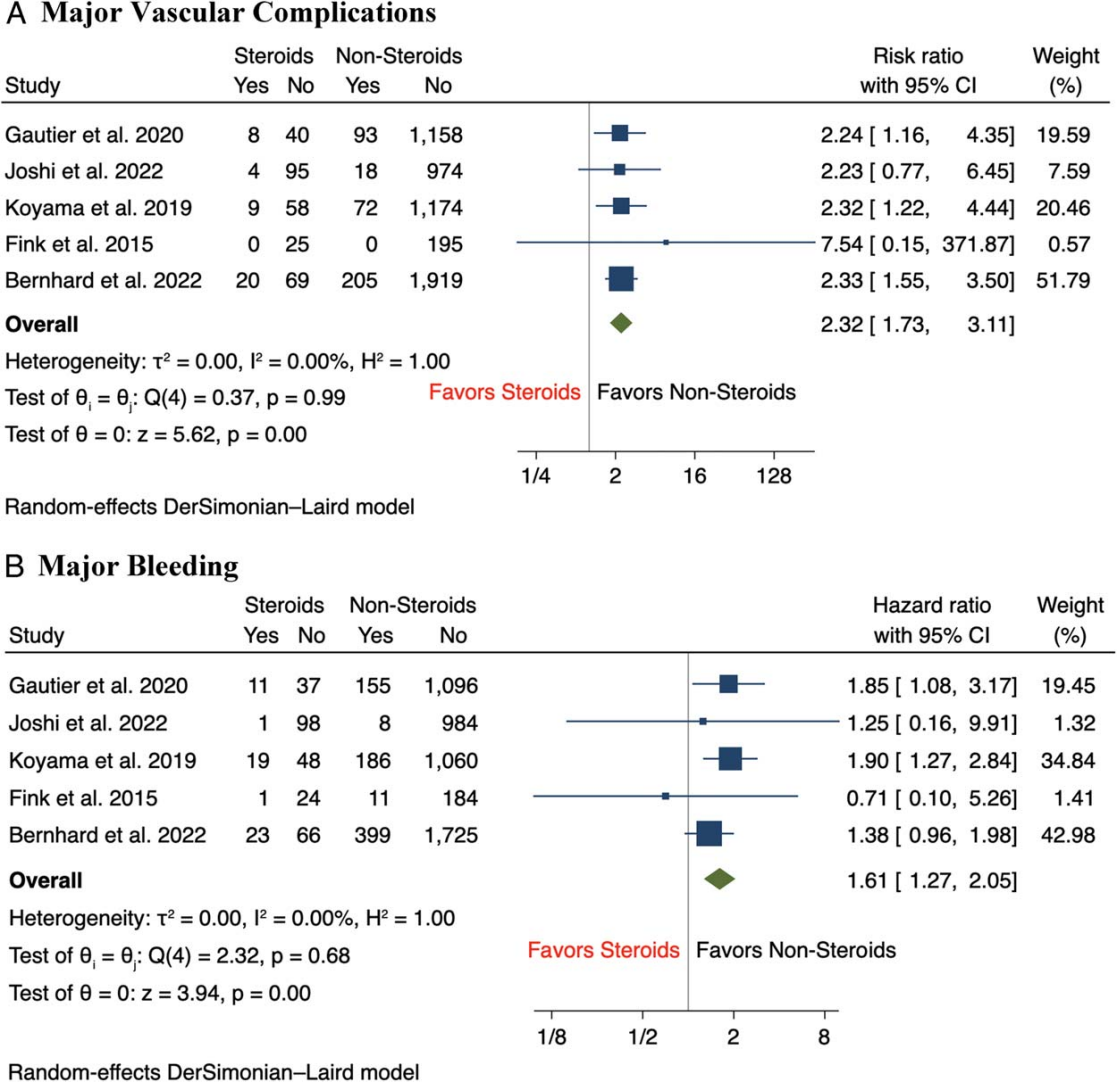
Forest plot of primary outcomes including (A) major vascular complications, (B) major bleeding.

**Figure 2:**
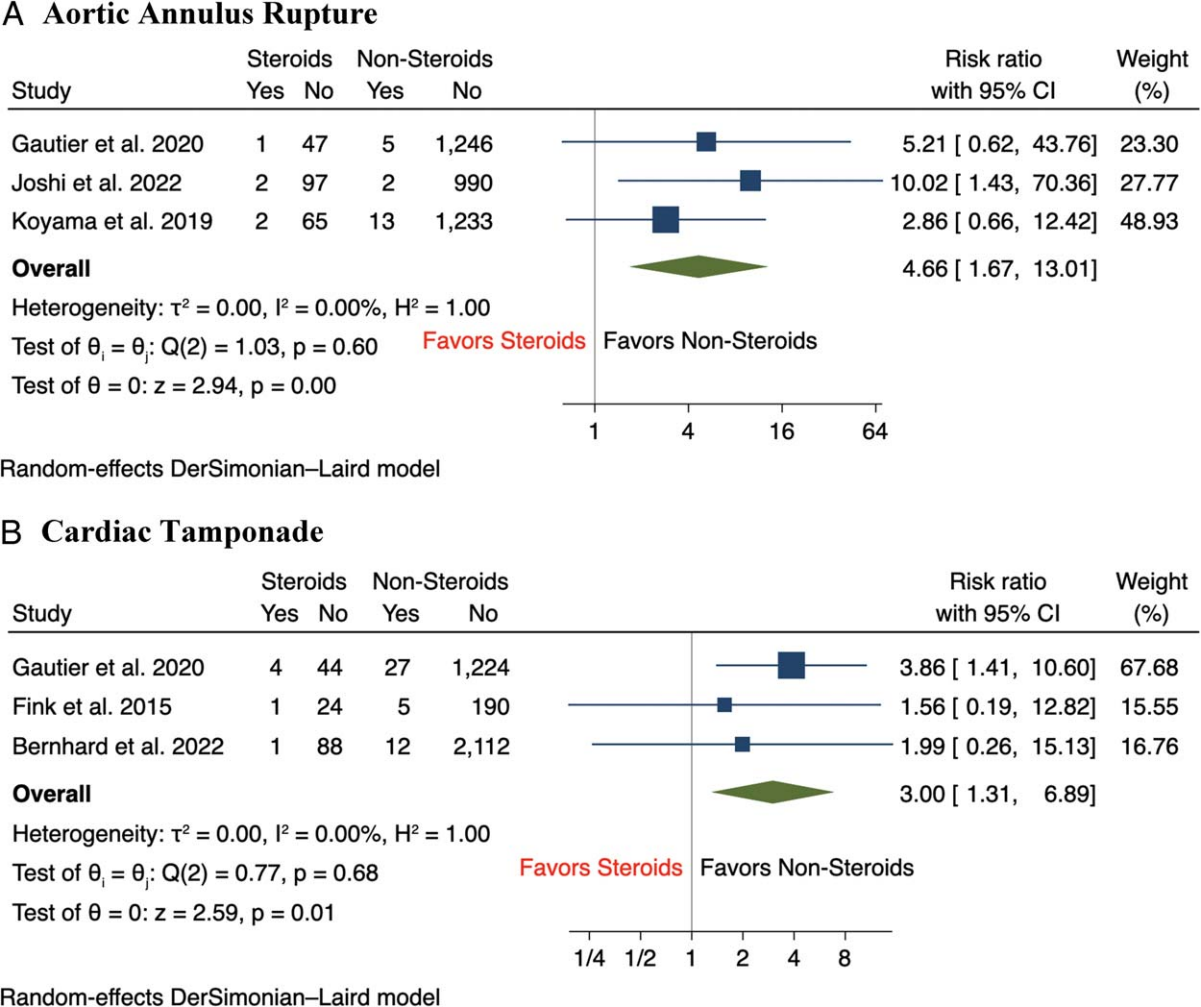
Forest plot of secondary outcomes including (A) aortic annulus rupture, (B) cardiac tamponade.

**Figure 3:**
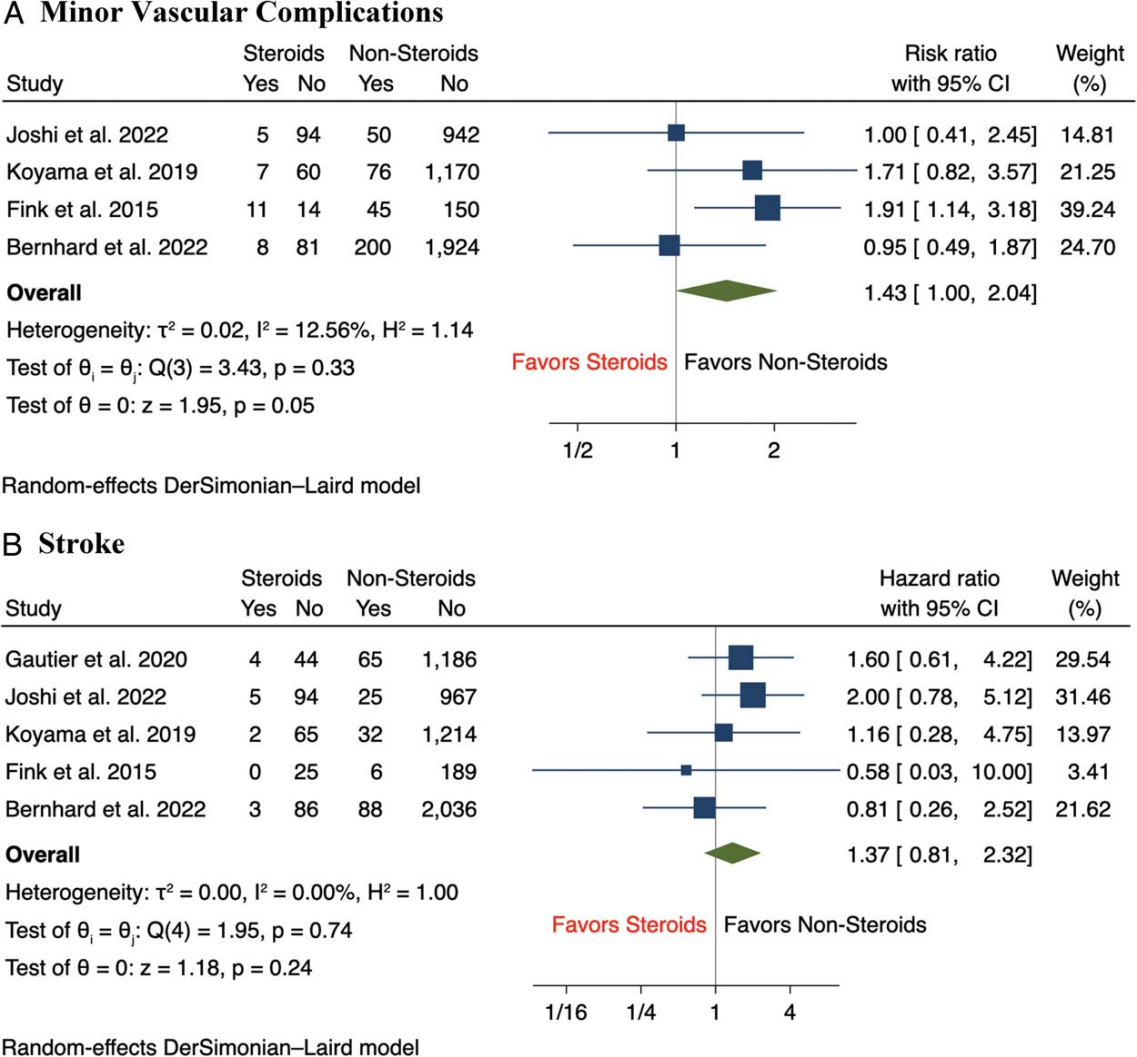
Forest plot of secondary outcomes including (A) minor vascular complications, (B) stroke.

**Figure 4:**
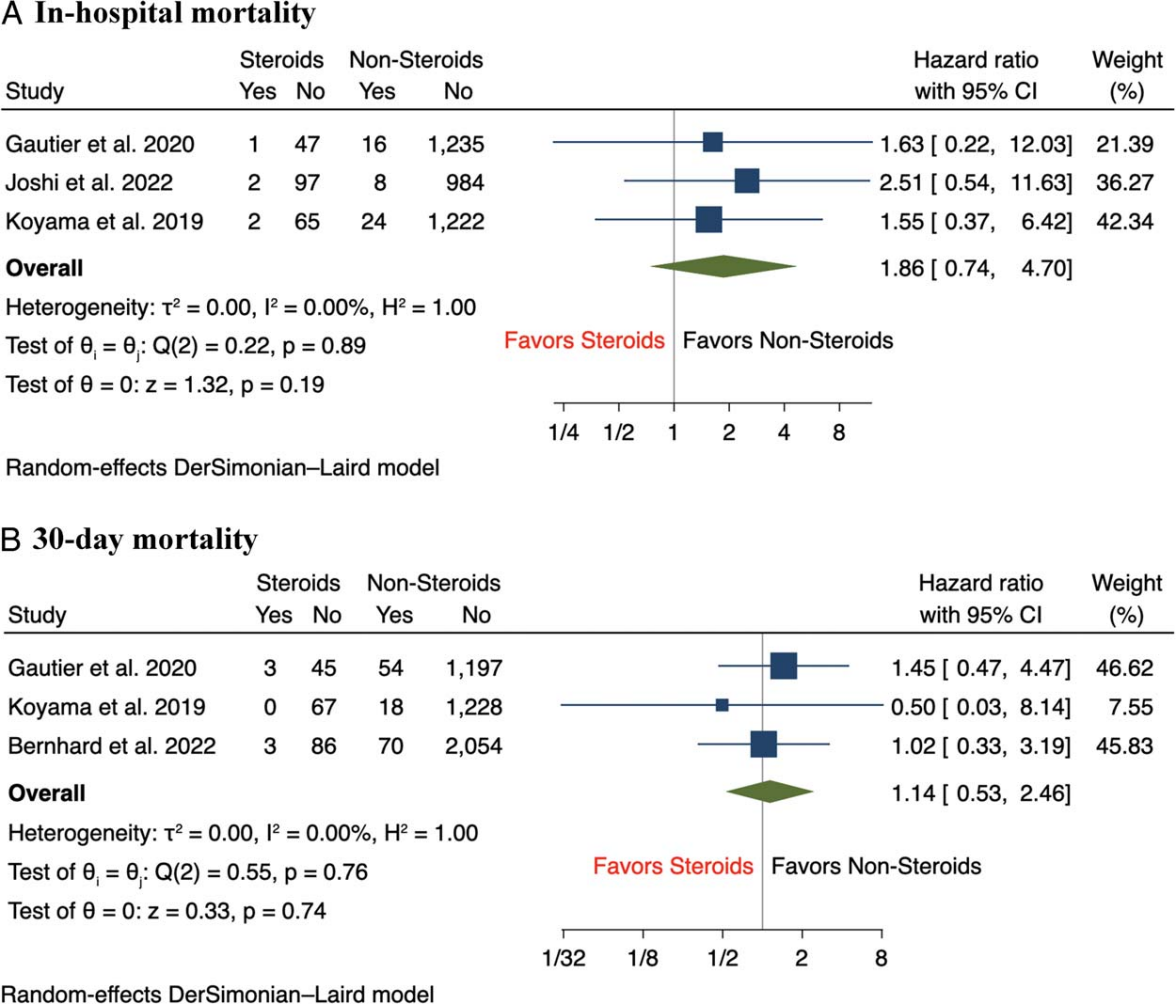
Forest plot of secondary outcomes including (A) in-hospital mortality, (B) 30-day mortality.

**Figure 5:**
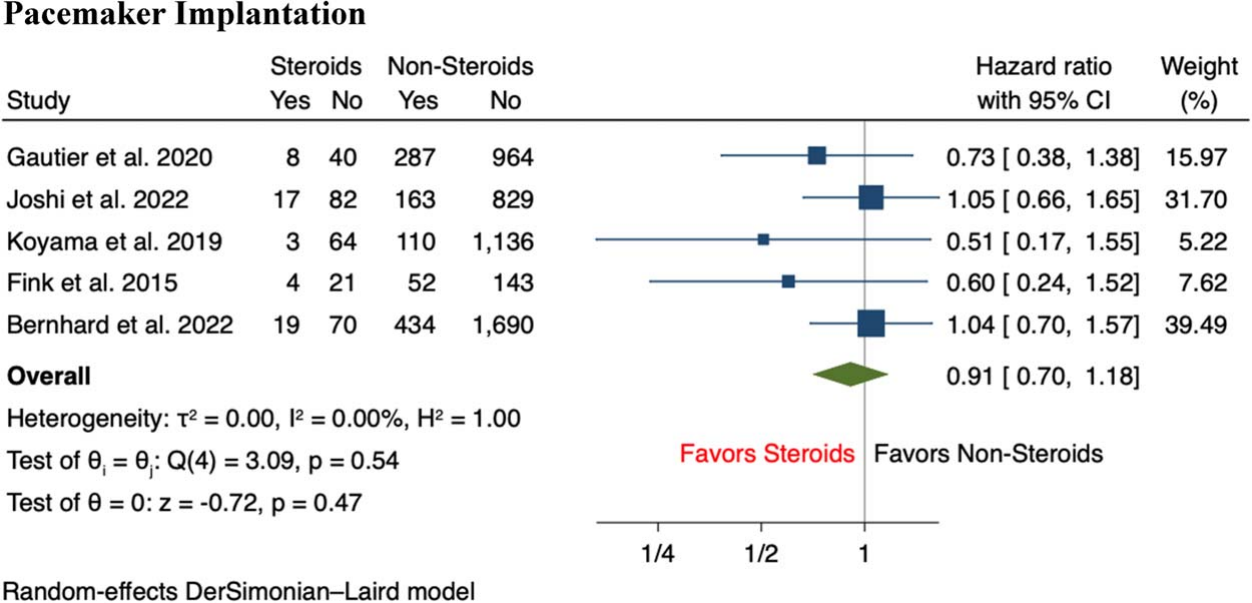
Forest plot of secondary outcomes including Pacemaker implantation.

Results of the meta-analysis showed that there was a statistically significant increase in the incidence of major vascular complications (12.5 vs 6.7%, RR 2.32, 95% CI: 1.73–3.11, *P*<0.001) and major bleeding (16.8 vs 13.1%, RR 1.61, 95% CI: 1.27–2.05, *P*<0.001) among CS group compared to nonsteroid groups, with minimal between-study heterogeneity across studies for both outcomes (*I* ^2^=0%) (Fig. 1A-B). In terms of secondary outcomes, the incidence of aortic annulus rupture (2.3 vs 0.6%, RR 4.66, 95% CI: 1.67–13.01, *P*<0.001) and cardiac tamponade (3.7 vs 1.2%, RR 2.79, 95% CI: 1.21–6.40, *P*=0.02) following TAVR were significantly higher in CS patients compared to those without CS use (Fig. 2A-B). Compared to patients without CS use, there was a nonsignificant trend of a higher incidence of minor vascular complications (11.1 vs 8.1%, RR 1.43, 95% CI: 1.00–2.04, *P*=0.05) and stroke (4.3 vs 3.7%, RR 1.37, 95% CI: 0.81–2.32, *P*=0.24) among patients with CS use (Fig. 3A-B). In terms of short-term mortality, both in-hospital mortality (2.3 vs 1.4%, RR 1.86, 95% CI: 0.74–4.70, *P*=0.19) and 30-day mortality (2.9 vs 3.1%, RR 1.14, 95% CI: 0.53–2.46, *P*=0.74) were comparable between both groups of patients (Fig. 4A-B). Lastly, there were no significant differences in the rate of pacemaker implantation following TAVR between both groups of patients (15.5 vs 18.0%, RR 0.91, 95% CI: 0.70–1.18, *P*=0.47) (Fig. 5).

### Sensitivity analyses

Sensitivity analyses were primarily conducted to test the robustness of primary analysis given the low heterogeneity observed across all outcomes. Sensitivity analyses were conducted for outcomes meeting the prespecified criteria, including major vascular complications, major bleeding, stroke, and pacemaker implantation. After implementation of the leave-one-out method, results were consistent with primary analysis, whereby the incidence of major vascular complications and major bleeding remained significantly higher among patients with CS use while the incidence of stroke and pacemaker implantation remained comparable between both groups of patients, confirming the robustness of results (Supplementary Figures 2–5, Supplemental Digital Content 5, http://links.lww.com/JS9/B802).

### Publication bias

Outcomes meeting eligibility for assessment of publication bias include major vascular complications, major bleeding, stroke, and pacemaker implantation. In addition, to minimal or no funnel plot asymmetry visualized, there was no evidence of publication bias through the quantitative Egger’s regression test with *P*>0.05 for the above outcomes (Supplementary Figures 6–9, Supplemental Digital Content 6, http://links.lww.com/JS9/B803).

## Discussion

The current meta-analysis quantitatively evaluated the adverse outcomes of TAVR patients on CS therapy. In our study, the incidence of major vascular complications and major bleeding were found to be significantly higher in patients with CS therapy compared to the control group, findings concordant with Koyama *et al*. and Gautier *et al*.^16,18^. Similarly, the incidence of aortic annular rupture following TAVR was found significantly higher in CS users in comparison to the nonsteroid user, supporting the findings of Joshi *et al*.^4^. However, post-TAVR stroke and 30-day mortality were comparable between the two groups, results concordant with the findings of Fink *et al*., and Bernhard *et al*.^17,19^. In addition, there was no significant difference in terms of in-hospital mortality between both groups of patients, a finding that was similar to a recently published nationwide analysis by Evbayekha *et al*.^20^.

The duration of steroid therapy was variable in different studies and ranged from >30 days to a mean duration of 1390 days of steroid therapy before TAVI^16,17^. On the other hand, the median daily prednisolone equivalent dose for the CS-treated group ranged from 5 mg to 7.5 mg. In these studies, corticosteroid (CS) therapy was used for various indications, including but not limited to polymyalgia rheumatica, giant cell arteritis, rheumatoid arthritis, renal or liver transplantation, and vasculitis^18,19^. In our study, the comorbidities were comparable between both the groups and the main comorbidities were HTN (84 vs. 85%) and DM (30 vs. 27%) in CS and nonsteroid groups, respectively.

A study conducted by Havakuk *et al*. in a small group of steroid-treated patients (3 days treatment) revealed no significant difference in major vascular complications, major bleeding, cardiac tamponade, minor vascular complications, stroke, and 30-day mortality following TAVR, suggesting that major complications may be related to the length of exposure to CS therapy^21^. Higuchi *et al*.^22^ studied the outcomes of TAVR and SAVR in steroid users and they found no significant difference between the two procedures in terms of major vascular complications, stroke, 30-day mortality, and life-threatening bleeding postprocedure. Similarly, Ellis *et al*. conducted a single-center observational study to examine the effect of CS therapy on the clinical outcomes of other interventional cardiac procedures, specifically PCI. They revealed that CS therapy was associated with a threefold increase in the risk of major vascular complications and a three to fourfold increase risk of coronary perforation in comparison to nonsteroid users. However, no significant difference was found in major ischemia events and mortality^6^.

In our study, we noted a higher incidence of major vascular complications, major bleeding, and aortic annular rupture in the CS group as opposed to the nonsteroid group. Despite these variances, the short-term mortality rates were comparable between the groups. Importantly, aortic annular rupture, a relatively infrequent but critical complication of TAVR, did not correspond to an increased mortality rate in the CS group. This discrepancy may be attributed to statistical factors. Specifically, the absolute effect size for these outcomes was more pronounced in the CS group, with a broad 95% CI and minimal heterogeneity across studies. The incidence of aortic annular rupture was 2.34% in the CS group versus 0.57% in the nonsteroids group, while in-hospital mortality rates were 2.34 and 1.38%, respectively. Essentially, the similar rates of aortic annular rupture and in-hospital mortality within the CS group, contrasted with a slightly higher mortality rate in the nonsteroids group, may have obscured a significant difference in mortality outcomes. Furthermore, the relatively smaller size of the patient cohort in the CS arm, compared to the nonsteroid group, could have impeded the detection of a significant difference in mortality rates. Therefore, if this trend persists, we expect that future studies with larger sample sizes, especially in the CS arm, might demonstrate a significant elevation in short-term mortality.

Corticosteroid use has been long known to cause vascular fragility by affecting collagen synthesis in the vascular wall and various cardiovascular diseases^23–26^. A multicenter study conducted by Pujades-Rudriguez *et al*.^27^ to evaluate cardiovascular complication in steroid user found that there is strong dose-dependent increases in hazards of all cardiovascular diseases, atherosclerotic diseases, atrial fibrillation, heart failure, and abdominal aortic aneurysm, regardless of the underlying immune-mediated disease, its activity, and duration. Thus, the effect of CS therapy on collagen synthesis by causing vascular fragility could be the proposed mechanism for having significantly higher major vascular complications, major bleeding, and aortic annulus rupture in the CS group as compared to nonsteroid controls in our study.

The strength of our study is that this is the most comprehensive meta-analysis conducted on a sample size, that is, 6136 patients (328 steroids, 5808 nonsteroids), to evaluate the vascular complications and outcome following TAVR on CS therapy. In our study, a significant post-TAVR difference was found in the incidence of major vascular complications, major bleeding, the incidence of aortic annulus rupture, and cardiac tamponade in CS user patients in comparison to the control. This study also has limitations that should be considered while interpreting the results. First, only five studies were included in the analysis and studies were largely observational in nature, thus the presence of confounding bias could not be ruled out. Furthermore, the small number of studies may be underpowered to detect a significant difference in the secondary outcomes such as the short-term mortality. Second, the number of CS patients was relatively smaller than the nonsteroid patients. While the baseline comorbidities appeared to be comparable between both groups, there may be other potential confounders that may explain the observed increase in the peri-procedural complications in these patients. Further sophisticated analytical methods to explore for potential effect modifiers including subgroup and regression analysis were not conducted given the lack of number of studies or respective covariates. Thus, it would be intriguing to observe the impact of these variables on the peri-procedural complications. Lastly, the information regarding the indication, dosage, and duration of corticosteroid (CS) administration was absent in some of the included studies. Consequently, the results are preliminary and should be interpreted with these limitations in mind. Further validation from large, multicenter cohort or randomized trials are warranted to elicit the true impact of CS therapy on TAVR.

## Conclusion

In this comprehensive meta-analysis, we observed a notable association between CS use and increased risks during TAVR. Specifically, patients in the CS group exhibited a statistically significant elevation in the incidence of major vascular complications and major bleeding compared to their nonsteroid counterparts. Secondary outcomes further revealed a heightened incidence of aortic annulus rupture and cardiac tamponade in CS patients. These findings underscore the necessity for meticulous clinical evaluation and patient counseling when considering TAVR in individuals on CS therapy.

## Ethical approval

Not applicable.

## Consent

Not applicable.

## Source of Funding

This research received no external funding.

## Author contribution

S.P.A. and J.E.C.: conceptualization; V.J.: methodology; J.E.C. and S.P.A.: data extraction and screening; S.P.A.: formal analysis and investigation; V.J., A.V., M.H., S.P.A., A.R., J.E. C., S.R., and J.I.: writing – original draft preparation; V.J., H.K., V.R.B., J.I., and A.B.S.: writing – review and editing.

## Conflicts of interest disclosure

The authors declare no conflicts of interest.

## Research registration unique identifying number (UIN)

1. Name of the registry: not applicable.
2. Unique identifying number or registration ID: not applicable.
3. Hyperlink to your specific registration (must be publicly accessible and will be checked): not applicable.

## Guarantor

Song Peng Ang.

## Data availability statement

Data is provided within the article and supplementary material.

## Provenance and peer review

Not commissioned, externally peer-reviewed.

## Acknowledgements

None declared by authors.

## Supplementary Material

SUPPLEMENTARY MATERIAL
