# Supplementary material for: Vascular complications and outcomes following transcatheter aortic valve replacement in patients on chronic steroid therapy: a meta-analysis
Source: Int J Surg. 2024 Feb 5;110(4):2421–9. doi: 10.1097/JS9.0000000000001132 (PMC11020145; doi:10.1097/JS9.0000000000001132)
Supplement: SUPPLEMENTARY MATERIAL [file js9-110-2421-s004.docx]

| Study | Selection | Comparability | Outcome | Total |
| --- | --- | --- | --- | --- |
| **Gautier et al (28)** | ** | ** | ** | 7/9 |
| **Joshi et al (12)** | ** | ** | ** | 6/9 |
| **Koyama et al (16)** | **** | ** | ** | 8/9 |
| **Fink et al (18)** | ** | ** | ** | 6/9 |
| **Bernhard et al (29)** | **** | ** | ** | 8/9 |
