# Supplementary material for: Vascular complications and outcomes following transcatheter aortic valve replacement in patients on chronic steroid therapy: a meta-analysis
Source: Int J Surg. 2024 Feb 5;110(4):2421–9. doi: 10.1097/JS9.0000000000001132 (PMC11020145; doi:10.1097/JS9.0000000000001132)
Supplement: SUPPLEMENTARY MATERIAL [file js9-110-2421-s005.docx]

**+**
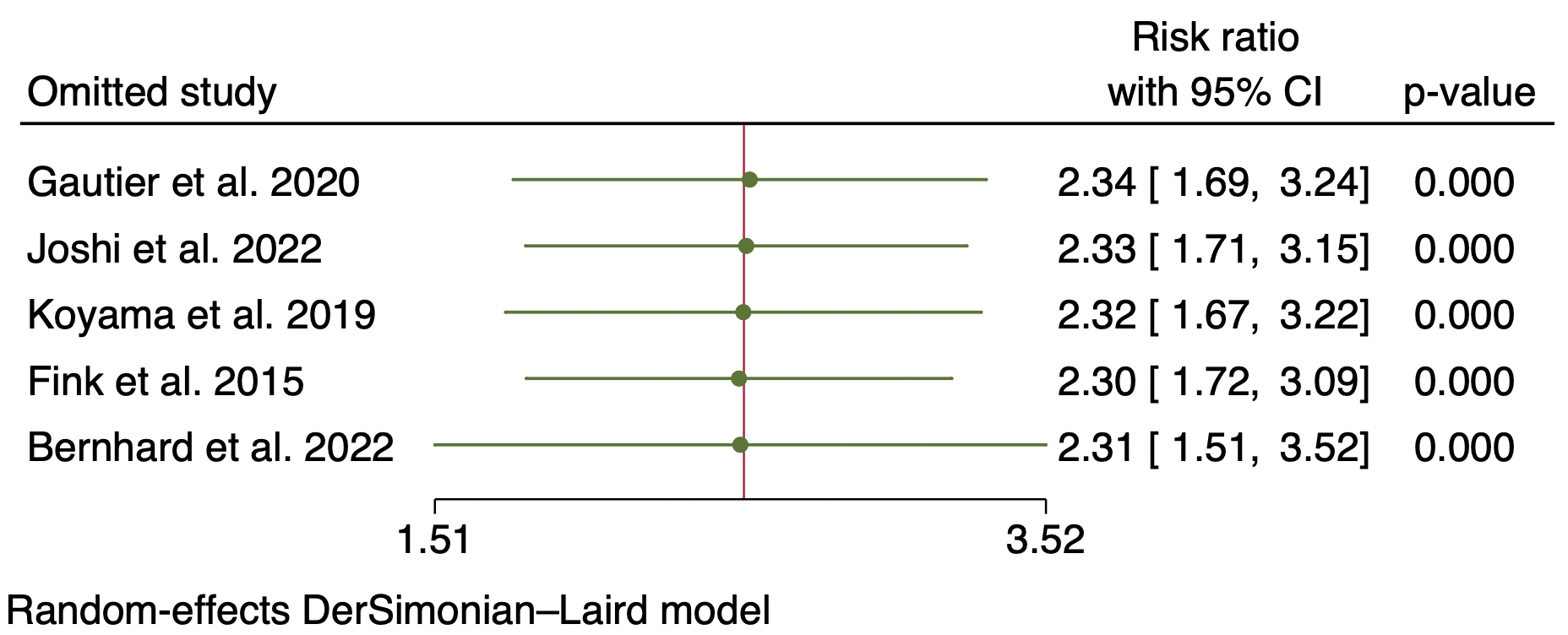


**Supplementary Figure 2** Leave-one-out analysis for major vascular complications


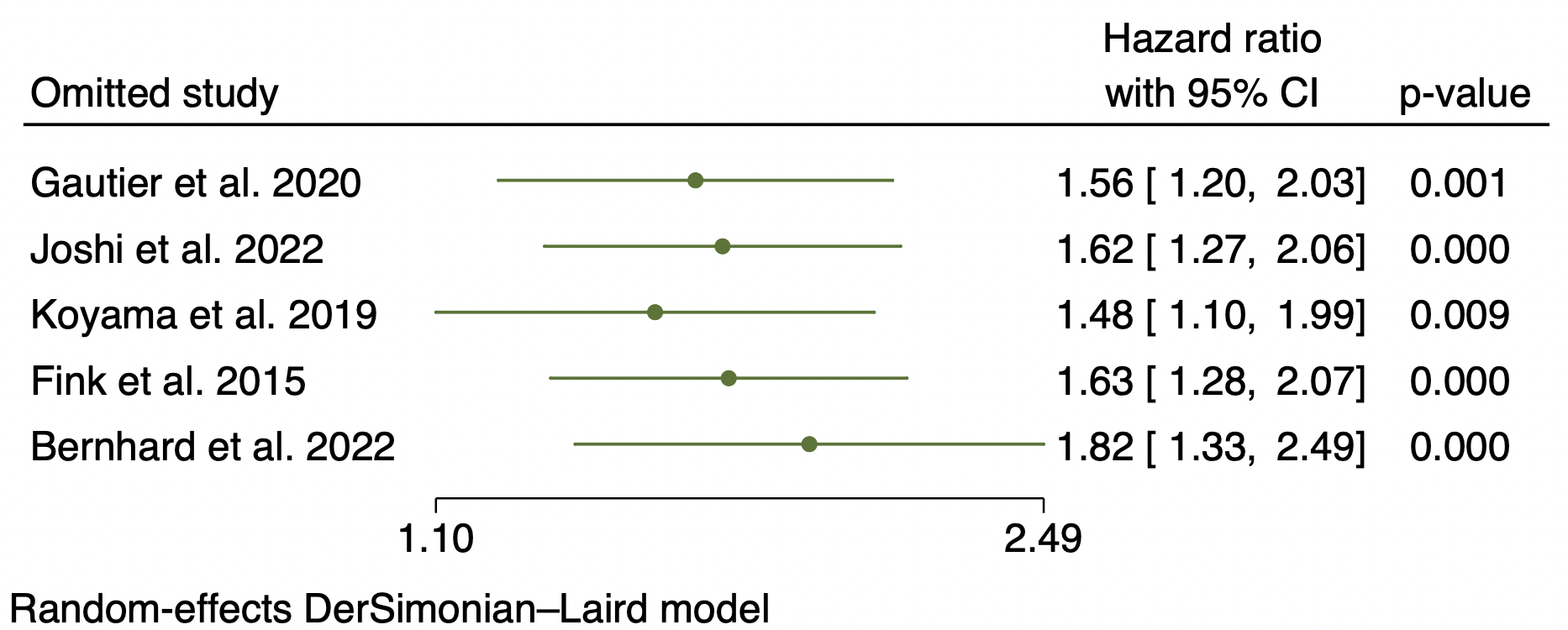


**Supplementary Figure 3** Leave-one-out analysis for major bleeding


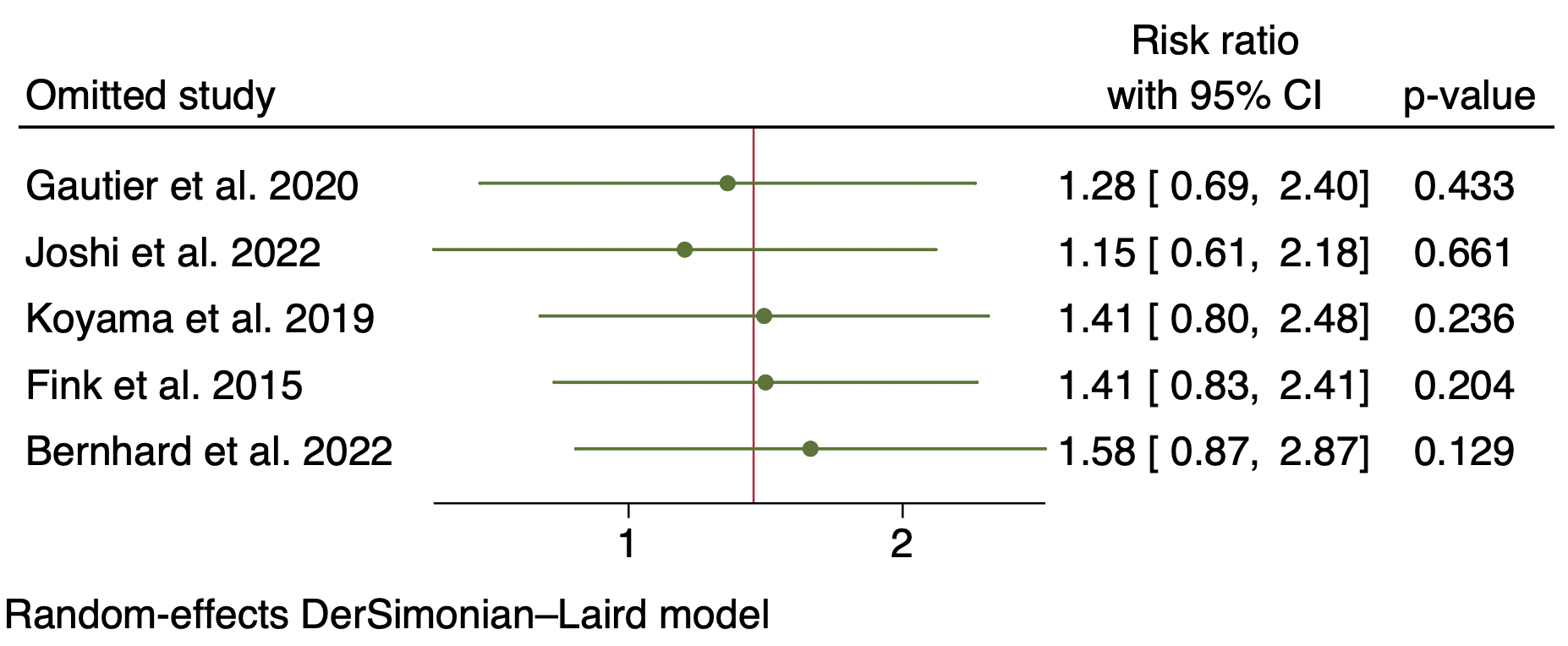


**Supplementary Figure 4** Leave-one-out analysis for stroke


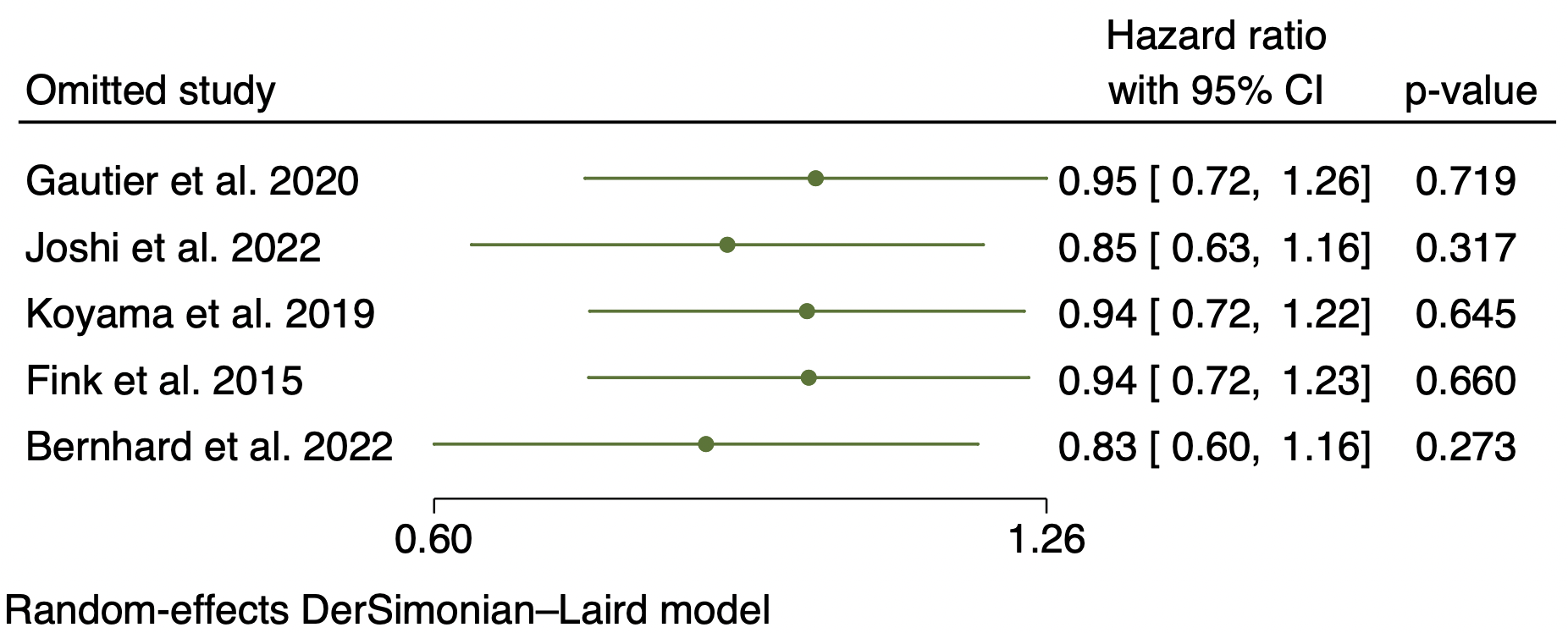


**Supplementary Figure 5** Leave-one-out analysis for pacemaker implantation
